# Dating Application Use and Its Relationship with Mental Health Outcomes Among Men Who Have Sex with Men in Urban Areas of Thailand: A Nationwide Online Cross-Sectional Survey

**DOI:** 10.3390/ijerph22071094

**Published:** 2025-07-09

**Authors:** Sarawut Nasahwan, Jadsada Kunno, Parichat Ong-Artborirak

**Affiliations:** Department of Research and Medical Innovation, Faculty of Medicine Vajira Hospital, Navamindradhiraj University, 681 Samsen Road, Dusit, Bangkok 10300, Thailand; 6701201009@nmu.ac.th (S.N.); jadsada@nmu.ac.th (J.K.)

**Keywords:** dating application, mental health, psychological distress, depression, men who have sex with men, urban area

## Abstract

Dating applications (DAs) are widely used to establish social and sexual connections among men who have sex with men (MSM), particularly in urban areas. In this study, we aimed to examine the associations between DA use and mental health among Thai MSM. An online cross-sectional survey was completed by 442 MSM residing in Bangkok and urban municipalities across all regions of Thailand. Psychological distress (PD) and probable depression were assessed using the General Health Questionnaire (GHQ-12) and the Patient Health Questionnaire (PHQ-9), respectively. Of the participants, 62.7% were current users, with 33.2% experiencing PD and 33.9% having depression. A logistic regression analysis showed that PD was significantly associated with late-night use (AOR = 2.02, 95% CI: 1.08–3.78), matching failure (AOR = 1.95, 95% CI: 1.12–3.38), rejection (AOR = 2.07, 95% CI: 1.18–3.62), and ghosting (AOR = 1.78, 95% CI: 1.02–3.11). Simultaneously, depression was significantly associated with using DAs with the motivation of hooking up (AOR = 2.27, 95% CI: 1.05–4.93), privacy violations (AOR = 2.76, 95% CI: 1.42–5.38), unsolicited sexual images (AOR = 2.04, 95% CI: 1.11–3.74), physical assault (AOR = 2.97, 95% CI: 1.57–5.61), harassment (AOR = 2.54, 95% CI: 1.37–4.70), scams (AOR = 2.59, 95% CI: 1.41–4.77), and extreme disappointment from DA use (AOR = 5.98, 95% CI: 1.84–19.41). These findings highlight how DA usage patterns and negative experiences may contribute to the poorer mental health among MSM in urban areas.

## 1. Introduction

Dating applications (DAs) are online smartphone platforms that provide individuals with the opportunity to build both platonic and romantic relationships quickly and enable connections based on shared interests, lifestyles, or activities [1,2,3]. Especially in urban areas characterized by high population density, individuals often struggle to meet and establish relationships due to the fast-paced lifestyle and increased competition in these environments. Consequently, DAs have become the easiest way to build relationships [4]. These factors have made DAs widely popular globally across all ages and backgrounds [3,5], particularly among men who have sex with men (MSM). Over half of MSM are frequent users who commonly use these platforms to seek sexual partners and expand their social networks [6,7,8].

According to a survey in Thailand, approximately 40% of the Thai population uses DAs, mostly comprising teenagers or young adults [9]. Usage was highest in Bangkok, Chonburi (Pattaya), and Chiang Mai [10,11]. Similarly to global trends, the use of DAs among MSM in Thailand is notably high. Approximately 73% of Thai MSM use DAs to seek partners and integrate DAs into their daily social lives [12]. These applications facilitate MSM in finding relationships that align with their desires, especially for sexual encounters [13,14]. While DAs offer opportunities for social connections, existing studies have also identified an association of DA use with negative health outcomes. These outcomes are linked to risky sexual behaviors, such as having multiple sexual partners, substance use, and an increased risk of sexually transmitted infections, particularly among MSM [6,7,8,12,14,15,16]. Moreover, DAs are also platforms that significantly contribute to the occurrence of violence and sexual harassment [17,18] and they raise several safety concerns, including the unauthorized sharing of personal information and money scams, especially for diverse gender group users [19,20]. Additionally, DAs are designed to focus on users’ appearances, which may create social pressure and idealistic values that lead users to be dissatisfied with their bodies and other mental health problems such as depression and psychological distress [21,22]. Previous empirical studies have highlighted further mental health impacts associated with DA usage, including reduced self-confidence as well as increased psychological distress and depression among MSM [23,24,25]. Users often experience loneliness and lower life satisfaction [23], while unsolicited sexting, harassment, and discrimination related to users’ appearance and identity significantly worsen mental health outcomes [24,26]. Collectively, these studies confirm a relationship between dating app use and adverse mental health outcomes in these populations.

Globally, mental disorders account for one in six years lived with disability, with worsening trends [27]. The social dynamics of urban environments, characterized by cultural diversity, increase the likelihood of conflicts, which further exacerbate negative psychological impacts [28,29]. In Thailand, the prevalence of depression in 2022 was reported at 19.4% [30]. Notably, in Bangkok, 54.36% of individuals were self-assessed as being at risk for depression [31]. MSM in Thailand face significant mental health challenges, such as psychological distress, depression, anxiety, and low self-esteem [32,33]. Despite growing societal openness about the diversity of sex, Thai MSM still deal with depression linked to discrimination, victimization, and concealment of their sexuality, as well as other social factors [34,35,36,37,38]. These issues may lead individuals to engage in sexual activities as a coping mechanism [39,40,41], and DAs effectively address this need [14]. These factors highlight the necessity of understanding DA usage and mental health problems among Thai MSM.

While research on the impact of DAs is abundant, few studies delve deeply into the unique characteristics, usage patterns, and user experiences specific to these platforms. In Thailand, most existing studies primarily focus on the impact of risky sexual behaviors and substance use, often neglecting the mental health issues associated with DA usage. This lack of comprehensive research on both mental health outcomes and DA use patterns among MSM limits our understanding of the issue and its broader implications. Furthermore, the cultural and social differences between countries suggest that existing findings may not be fully applicable to the Thai context.

Thus, in this study, we aim to examine DA use and the prevalence of mental health issues, and the relationship between them, among MSM in urban areas of Thailand. We hypothesize that certain dimensions of DA use are associated with poorer mental health, as indicated by elevated levels of psychological distress and depression. These dimensions include a history of DA usage; specific usage patterns (e.g., the number of apps used, frequency, duration, and timing); user motivations (e.g., making friends, relieving loneliness, hooking up, or seeking a long-term relationship); and DA-related experiences (e.g., matching failure, ghosting, rejection, privacy violations, physical assault, money scams, and overall disappointment). The findings provide comprehensive information that can inform the development of effective mental health policies and support systems for MSM in Thailand.

## 2. Materials and Methods

### 2.1. Study Design

An online cross-sectional survey was conducted using Google Forms. Data collection took place between March and April 2025 through social media, including Facebook groups (e.g., “Thailand LGBTQ + Community”, “Gay dating groups”), Telegram, and other online communities. Furthermore, the survey link was shared through non-governmental organizations (NGOs) across all regions of Thailand, including the Rainbow Sky Association of Thailand, M-Plus Thailand, and SWING via the NGOs’ websites and announcement boards at the service center.

### 2.2. Participants

Convenience sampling and snowball sampling methods were used to recruit participants. Participants were able to access the survey by scanning the QR code on the invite poster or clicking the link on the online post. They were informed that their responses would be anonymous. To prevent duplicate responses, participants’ email addresses were used solely for verification purposes. While no other use of email occurred, the use of email communication may have raised privacy concerns; therefore, all efforts were made to ensure confidentiality and secure handling of contact information. Email data were anonymized and stored securely, and all email addresses were deleted immediately after confirming the absence of duplicate entries. Upon entering the survey page, information regarding the study’s objective and confidentiality was provided, and participants were given the option to select “agree” or “do not agree” to participate before starting the questionnaire. Choosing “agree to participate” constituted implied consent, allowing participants to continue with the questionnaire. Eligible participants were Thai males aged 18 or older who had had sexual activity with other men, regardless of their sexual orientation. Participants also had to live in urban areas in Thailand, such as Bangkok and other cities or urban municipalities across all six regions: northern, northeastern, central, eastern, western, and southern. They had to be able to read Thai and to not be receiving treatment or medication for any psychiatric conditions at the time of completing the survey.

The sample size was calculated using Cochran’s formula for a single population proportion [42], with a 95% confidence level and a 5% margin of error. An estimated proportion (*p*) of 0.50 was used to maximize the sample size. To account for incomplete data, the sample size was increased by 15%, resulting in a total target of 444 participants. Of these, 442 participants with complete data were included in the final analysis.

### 2.3. Measurements

Data were collected using a self-reported online questionnaire, which was divided into three sections: demographic and health-related information, DA use, and mental health outcomes.

Firstly, demographic and health-related information measures included age, sexual orientation, relationship status, education level, income, occupation, and residential area (specific urban area). It also included self-rated measures of family relationship level, body image satisfaction, physical health satisfaction, and levels of perceived social stigma, sexual history and risky sexual behaviors in the previous 6 months (e.g., number of sex partners, condom use, group sex participation, alcohol drinking, smoking, substance use and recreational drugs (poppers) during sex), and history of HIV testing in the past 6 months.

The second section focused on the characteristics, patterns, motivations, and experiences related to DA use. Participants were asked about their DA use history and were classified into three groups based on their responses: ‘Current Use’ (those who had used DAs within the past six months), ‘Ever Used’ (those who had used DAs previously but not within the past six months), and ‘Never Use’ (those who had never used DAs). Participants who selected ‘Ever Used’ or ‘Never Use’ were directed to skip the questions related to DA use and move directly to the mental health outcome section. The questionnaire included DA preferences, the number of DA uses, the duration since first use, the frequency of DA use, the average time spent per session, the most days and times they typically used DAs, disclosure, motivations for DA use, negative experiences through DA use, the number of partners met face to face through DA, and overall disappointment from DA use.

Finally, mental health outcomes were measured using validated and developed instruments to suit the context of Thailand, including the Thai version of the 12-item General Health Questionnaire (Thai GHQ-12) for assessing psychological distress [43,44] and the Thai version of the Patient Health Questionnaire-9 (Thai PHQ-9) for assessing depressive symptoms [45,46]. The Thai GHQ-12 is a self-report questionnaire consisting of 12 questions related to feelings and behaviors over the past 2–3 weeks. Respondents have to choose the answer that reflects their feelings the most. Each question has four response items, with scores of 0, 0, 1, and 1 assigned to each item in that order, following the GHQ scoring method. The total score is interpreted, with a cutoff point of two or more indicating psychological distress. The Cronbach alpha coefficient is 0.86, with sensitivity of 0.78, and specificity of 0.85 [44]. The Thai PHQ-9 consists of 9 questions about the frequency of depressive symptoms occurring over the past two weeks. Respondents must choose the answer that best reflects their feelings the most. Each question has four response items, with scores ranging from 0 to 3. The total score is interpreted, with a cutoff point of 9 or more indicating probable depression. The Cronbach alpha coefficient is 0.79, with a sensitivity of 0.84 and a specificity of 0.77 [46]. For assessing depression severity, the scores of 5, 10, 15, and 20 correspond to mild, moderate, moderately severe, and severe depression, respectively [47].

### 2.4. Statistical Analysis

Statistical analysis was performed using SPSS software version 29.0, with the significance level set at *p* < 0.05. Descriptive statistics were used to describe participants’ demographic and health-related information, DA use, and mental health outcomes. Frequencies and percentages were used to describe categorical variables, while means, standard deviations (SDs), minimum (min) and maximum (max) values, medians, and interquartile ranges (IQRs) were used for continuous variables. For mental health outcomes, data were dichotomized into ‘no’ or ‘yes’ categories indicating the presence of psychological distress and probable depression, based on cutoff points reported in prior research [44,46]. Chi-square tests were conducted to examine demographic and health-related variables associated with mental health outcomes. Kruskal–Wallis tests were used to compare GHQ-12 and PHQ-9 scores across different DA usage groups (current use, ever used, never used) due to the data not following a normal distribution. Subsequently, binary logistic regression was performed to analyze the relationship between certain dimensions related to DA use and mental health outcomes among participants who were current users. Univariable analyses were used to estimate crude odds ratios (CORs) with 95% confidence intervals (CIs), while multivariable logistic regression was used to calculate adjusted odds ratios (AORs) with 95% CIs after controlling for demographic and health-related variables that showed significant associations with the outcome (*p* < 0.05) in the chi-square analysis. To avoid multicollinearity and maintain statistical power, each independent variable representing patterns, motivations, and experiences related to DA use was tested separately in regression models. All variance inflation factor (VIF) values were below 2, indicating no evidence of multicollinearity. Multilevel analysis with random effects for Thai regions was not used due to very low intraclass correlation coefficients (ICCs < 0.01).

## 3. Results

### 3.1. Mental Health Outcomes

One in three of the participants (32.1%) was found to exhibit psychological distress, and approximately one-third (33.5%) exhibited probable depression. When considering the depression severity at five levels, 43.2% of the participants reported no to minimal symptoms, followed by mild (33.3%), moderate (13.1%), moderately severe (7.7%), and severe symptoms (2.7%).

### 3.2. Demographic and Health-Related Information

The majority of the participants, 54.3%, were aged between 26 and 35 years, followed by 27.4% who were aged 18 to 25 years [median (IQR) = 29 (9)]. Most of the participants identified as gay (79.6%) and were single (60.9%). A significant portion of the participants held a bachelor’s degree (66.1%), worked in the private sector (46.2%), and earned between THB 15,001 and 29,999 per month (36.2%) [median (IQR) = THB 20,000 (15,000)]. Additionally, 45.5% of the participants resided in Bangkok. The family relationship level was rated as very good by 45.9%, and the participants reported good satisfaction with their body image (45.7%) and similarly for their physical health (45.0%). A significant proportion (45.9%) reported high levels of perceived social stigma. Regarding risky sexual behaviors, 85.1% of the participants had engaged in sexual activity in the past six months. Among the sexually active participants (*n* = 376), 62.8% reported having multiple sexual partners, while only 35.9% consistently used condoms during sex. Approximately 46.5% of the participants engaged in group sex, 47.6% consumed alcohol, 20.5% smoked during sex, 12.8% used substances, and 46.8% used recreational drugs before or during sex. In terms of the health-related information, 67.7% of the participants (*n* = 434) had undergone HIV testing, as shown in Table 1.

Chi-square tests were initially conducted to examine the relationships between the demographic and health-related variables and mental health outcomes. The variables that were significantly associated with psychological distress included family relationships (*p* < 0.001), body image satisfaction (*p* < 0.001), physical health satisfaction (*p* < 0.001), number of sexual partners (*p* = 0.045), and group sex participation (*p* = 0.005). Regarding depression, significant associations were found with age (*p* = 0.043), education (*p* = 0.041), family relationships (*p* = 0.030), body image satisfaction (*p* < 0.001), physical health satisfaction (*p* < 0.001), perceived social stigma (*p* = 0.021), and smoking before or during sex (*p* = 0.020).

### 3.3. DA Usage Characteristics

Of the participants, 12.2% reported never having used DAs, 25.1% identified as former users, and the majority (62.7%) were current users, as shown in Table 2. Of these current users (*n* = 277), the most used DAs were Tinder (78.7%), followed by Hornet (75.7%), Blued (54.2%), Facebook Dating (53.4%), Grindr (43.3%), Jack’d (34.7%), Omi (34.3%), and Coffee Meets Bagel (16.2%). Over half of the current users (54.5%) reported using more than three different DAs, and 78.0% had been using DAs continuously for two years or more. Daily usage was reported by 43.3% of the participants, with 70.0% spending less than 20 min per session. Most of the participants (67.5%) reported their highest usage on weekends (Friday to Sunday), with peak usage times between 18:01 and 22:00 (84.8%) and between 22:01 and 02:00 (73.6%). The primary motivations for using DAs included relieving loneliness (82.3%), making new friends (78.3%), and finding casual sexual partners (79.4%). The most common interaction methods included sending images or videos (81.9%), video calling/FaceTime (33.6%), buying or selling services (16.6%), and live streaming (10.8%). The most frequently disclosed personal details were face pictures (86.3%) and real nicknames (59.6%).

In terms of negative experiences with DA use, the most frequently reported were being ghosted (50.2%), failing to find matches that met their preferences (46.2%), and being ignored or rejected (41.2%). Additionally, 41.2% reported experiencing sexual harassment, and 61.4% had received unsolicited sexual images. Regarding the number of partners met in person during the past six months, most participants (42.2%) reported having one to two partners, followed by 24.5% who reported having three to five partners. A majority also expressed disappointment with DA use, with 31.4% reporting moderate and 24.2% reporting high levels of disappointment, as shown in Table 3.

### 3.4. Association Between DA Use and Mental Health Outcomes

When the mental health outcomes were compared across the current users, ever users, and never users of DAs, as shown in Table 2, no statistically significant differences were found in either psychological distress or depression. The Kruskal–Wallis test indicated no significant differences in the GHQ-12 scores among the current users (1.6 ± 2.5), ever users (1.6 ± 2.6), and never users (1.6 ± 2.7) (*p* = 0.946). Similarly, no statistically significant differences were found in the PHQ-9 scores, with mean scores of 6.7 ± 6.0 for the current users, 6.4 ± 5.5 for the ever users, and 5.8 ± 5.6 for the never users (*p* = 0.660).

Among the current users, the associations between DA use—in terms of patterns, motivations, and experiences—and mental health outcomes, as analyzed by binary logistic regression, are presented in Table 3. Regarding the usage patterns, using more than three dating applications (COR = 1.81, 95% CI = 1.08–3.02) and app usage during late-night hours (COR = 2.09, 95% CI = 1.17–3.76) were significantly associated with psychological distress. After adjusting for demographic and health-related covariates, only late-night app usage remained significantly associated with psychological distress (AOR = 2.02, 95% CI = 1.08–3.78). However, no variables were significantly associated with depression.

Regarding the motivations and experiences with DA use, using DAs with the motivation of hooking up (COR = 2.14, 95% CI = 1.07–4.29), frequently failing to find suitable matches (COR = 2.28, 95% CI = 1.37–3.80), being ignored or rejected (COR = 2.25, 95% CI = 1.35–3.75), and being ghosted (COR = 2.05, 95% CI = 1.23–3.41) were all significantly associated with psychological distress. In the adjusted model, hooking up was no longer significant. The participants who frequently failed to find suitable matches had 1.95 times higher odds of experiencing psychological distress (AOR = 1.95, 95% CI = 1.12–3.38) compared to those with infrequent or no such experiences; similarly, those who were often ignored or rejected had more than twice the odds (AOR = 2.07, 95% CI = 1.18–3.62), and those who frequently experienced ghosting had 1.78 times higher odds (AOR = 1.78, 95% CI = 1.02–3.11).

For depression, several significant factors were identified: using DAs with the motivation of hooking up (COR = 2.23, 95% CI = 1.11–4.46), seeking long-term relationships (COR = 1.86, 95% CI = 1.08–3.19), or sexual services (COR = 1.96, 95% CI = 1.04–3.66); experiencing privacy violations (COR = 2.81, 95% CI = 1.54–5.13); receiving unsolicited sexual images (COR = 1.93, 95% CI = 1.13–3.29); being physically assaulted by someone met through the app (COR = 2.56, 95% CI = 1.53–4.29); experiencing sexual harassment or coercion (COR = 2.11, 95% CI = 1.27–3.51); falling victim to a money scam (COR = 2.12, 95% CI = 1.28–3.53); and reporting the highest level of disappointment from DA use (COR = 5.70, 95% CI = 1.97–16.47). In the adjusted model, the motivations of seeking long-term relationships and sexual services were no longer significant. However, using DAs for hooking up remained significantly associated with depression (AOR = 2.27, 95% CI = 1.05–4.93), as did experiencing privacy violations (AOR = 2.76, 95% CI = 1.42–5.38), receiving unsolicited sexual images (AOR = 2.04, 95% CI = 1.11–3.74), being physically assaulted (AOR = 2.97, 95% CI = 1.57–5.61), experiencing sexual harassment (AOR = 2.54, 95% CI = 1.37–4.70), falling victim to scams (AOR = 2.59, 95% CI = 1.41–4.77), and reporting extreme (AOR = 5.98, 95% CI = 1.84–19.41) or high (AOR = 2.71, 95% CI = 1.03–7.13) levels of disappointment compared to no disappointment.

## 4. Discussion

The current study presents findings on the DA use among urban MSM. The finding that 62.7% of the participants reported DA use within the past six months indicates the widespread adoption of online platforms for relationship-seeking. However, this proportion is lower than that reported in Boonchutima et al.’s study [48], which may reflect different dynamics in user behavior or the rise of alternative platforms like TikTok and Twitter (X).

In terms of the user characteristics, most of the users were aged 18–35 and had at least a bachelor’s degree, suggesting that young and educated individuals are more likely to use such technologies. The majority identified as gay and single, supporting findings from previous studies [13,49]. Interestingly, some partnered individuals also used DAs, reflecting diverse usage motivations beyond romantic or sexual objectives. Moreover, over half of the users reported using more than three apps concurrently, highlighting a multi-platform strategy to increase matching opportunities. Tinder, Hornet, and Blued were the most commonly used platforms. This partially aligns with previous studies in Thailand [14,48], which reported Hornet, Jack’d, and Grindr as popular among MSM. The popularity of Tinder in the present study may reflect a shift toward more mainstream platforms with broader user bases. Additionally, the relatively high use of Blued may suggest increasing interest in DAs that offer social features beyond dating, such as live streaming and community interaction.

In addition, most of the participants had been using DAs for more than two years, reported frequent daily use, and preferred to use the apps during evenings and weekends, particularly between 18:00 and 22:00. These patterns are in line with previous findings [13,14,48]. However, the present study diverges from Goedel and Duncan’s study [49], which reported that users were more likely to use DAs on weekdays. Despite this difference, all the studies suggest frequent usage, particularly during non-working hours; this may indicate that MSM prefer to engage with these platforms during leisure time.

The DA users primarily reported motivations such as relieving loneliness, making friends, and hooking up. While seeking sexual partners remains a primary motive, as shown in previous studies [13,48,49], the high prevalence of social motivations also aligns with the conclusions of Thunyapipat et al.’s study [14]. This finding indicates that DAs satisfy not only sexual needs in MSM but also emotional and social needs.

Importantly, the present findings reveal that negative experiences are common among MSM users. The most frequently reported were ghosting, failing to find suitable matches, and being ignored or rejected. Many users also reported experiences of sexual harassment or coercion. These findings align with Echevarria et al.’s study [17], suggesting that DAs may expose users to harmful experiences such as rejection or harassment. Repeated exposure to these experiences can negatively affect individuals’ emotional and mental health, particularly among frequent or long-term users [21,22]. Although many users reported using DAs for casual sex, a significant number had only met 1–2 partners in person. This finding suggests that transforming online conversations into real-life meetings can be challenging. In addition, feelings of disappointment with DAs were also common, with 24.2% reporting high levels and 9.0% reporting extreme levels. This dissatisfaction may result from recurring negative experiences. It also highlights the importance of understanding how dating app usage relates to sexual behavior and broader health risks.

Regarding the prevalence of mental health outcomes, about one-third of urban MSM in this study experienced psychological distress and reported probable depression, representing a substantial proportion. This finding aligns with Thunyapipat et al.’s study [14], which reported that approximately one-third of MSM in Thailand were at risk of depression or other mental health conditions. In addition, the study by Encina et al. [34] found that the prevalence of depression among Thai MSM exceeded 50%, while Kittiteerasack et al. [36] reported that 40.3% of Thai LGBT adults met the criteria for depression. Although the prevalence in the present study was lower, it remains concerning and consistent with the elevated mental health burdens found among sexual minority groups in Thailand.

At the regional level, Tan et al. [50] found that the depression prevalence among LGBTQ populations in Southeast Asia ranged from 23.5% to 40.3%. The prevalence found in the present study falls within this range, reinforcing the broader trend of elevated mental health risks among sexual minorities in the region. Compared to findings in China, where the prevalence of depression among MSM was reported at 43.2% [51], the prevalence found in this study was slightly lower. These differences may reflect variations in sociocultural environments and mental health awareness, suggesting that differences in cultural norms regarding the emotional expression of MSM may also play a role. Additionally, discrepancies in the findings across studies may partly result from the use of different measurement tools or screening criteria for psychological distress and depression. Furthermore, the Minority Stress Theory [52] helps contextualize these results. MSM are often exposed to unique stressors such as stigma, discrimination, and the pressure to conceal their sexual orientation. While these stressors may not affect all individuals equally, they may contribute to elevated rates of psychological distress. 

The present study provides a comprehensive description of the relationship between DA usage and mental health outcomes among MSM in urban settings. While the initial analyses did not show a statistically significant relationship or differences regarding psychological distress or probable depression, the additional analyses revealed that the specific characteristics of DA usage and user experiences play a more critical role in mental health than the status of being a current user or non-user. This may indicate that the usage status alone does not adequately reflect the mental health impact of DAs. Individuals use dating apps for diverse purposes and experience both positive and negative interactions. While some users may engage with DAs in ways that support social connection by making friends or relieving loneliness, others may encounter negative experiences, such as rejection, harassment, or unsolicited sexual content. These differing experiences may obscure underlying associations when the participants are grouped only by their usage status. These findings correspond with those of Echevarria et al. [17] and Holtzhausen et al. [21], who emphasized that adverse mental health outcomes are more closely linked to how individuals use DAs, including the use frequency, motivations, and negative experiences, rather than the mere act of using them.

This study demonstrated that specific patterns, motivations, and experiences of DA use were significantly associated with psychological distress and depression. This finding expands upon previous studies that focused mainly on usage status. Notably, DA use during late-night hours was significantly associated with a twofold increase in the odds of experiencing psychological distress compared to use during other times of day. This may indicate that individuals experiencing stress or loneliness may be more likely to use apps during these hours or that late-night app usage may disrupt sleep cycles, subsequently increasing the risk of mental health problems [53]. Additionally, nighttime use may not only be a contributing factor but also a behavioral response to pre-existing emotional distress, where individuals turn to digital platforms as a means of distraction or temporary emotional relief.

This study also found that the motivations behind DA use significantly affect mental health. In particular, using DAs for hooking up was associated with more than twice the odds of experiencing depression. This may align with previous studies [13,48,49] that examined the psychological effects of engaging in casual sexual encounters or using dating applications primarily for sexual purposes. However, these studies did not explicitly investigate the link between such motivations and depression. Therefore, the present study offers new insights into this association, which may reflect underlying mechanisms such as emotional disengagement, unmet expectations, or frequent negative experiences [17]. Among individuals who seek connection or intimacy, the absence of emotional reciprocity in casual encounters may contribute to increased vulnerability to depressive symptoms. However, it is also possible that individuals experiencing psychological distress or depressive symptoms may turn to DAs as a strategy for coping with this mental health problem.

While usage patterns such as nighttime use and use with the motivation of hooking up were significantly associated with psychological distress or depression, other usage patterns (e.g., the number of DAs used, app usage frequency, and duration of overall use) did not show significant associations with mental health outcomes. This finding suggests that the intensity of use alone may be less relevant than the specific purposes and experiences related to DA use. Therefore, future research should consider both the intensity of DA use and the underlying behavioral and emotional dimensions, as these aspects may interact to influence mental health outcomes.

Furthermore, this study demonstrated a strong association between negative experiences and both psychological distress and probable depression. Users who frequently failed to find matches, experienced ghosting, or were ignored or rejected were approximately twice as likely to experience psychological distress. Additionally, experiences such as privacy violations, unsolicited sexual image sharing, sexual harassment, and money scams were associated with over twice the odds of depression compared to those of the participants without experiences of a specific negative event, with physical assault showing the highest AOR (2.97). These findings are consistent with a prior study showing that negative experiences with DAs, such as rejection, ghosting, or harassment, can deeply impact users’ mental well-being. Because many DAs emphasize appearance-based interactions, failing to find a match or being frequently ignored may lead users to internalize feelings of inadequacy or low self-esteem. Over time, these emotional effects may build up and increase the risk of depression [54,55,56].

Additionally, being exposed to more serious forms of harm like non-consensual image sharing or physical assault may directly trigger or worsen depression [17,19,20,57]. Specifically, experiencing physical assault may undermine an individual’s self-esteem and reduce their sense of control over their life. It may also contribute to loneliness, resulting from social withdrawal or feelings of isolation—particularly when individuals distance themselves from others to prevent further harm—ultimately increasing the risk of mental health problems in the long term due to these psychological responses [17]. A previous study found that many LGB adults are exposed to unsolicited explicit messages, offensive name calling, and scams while using online dating platforms, leading to detrimental effects on their mental health and well-being [58]. Another study found that non-consensual sexting significantly worsens mental health outcomes, such as depression, among transgender and gender-diverse adults [24]. Importantly, the users who reported high levels of disappointment had nearly threefold higher odds of experiencing depression, while those with extreme disappointment had an almost sixfold increase in this risk, respectively. This association may reflect the cumulative effects of unmet expectations, emotional exhaustion, and repeated exposure to rejection. When negative outcomes occur repeatedly despite effort or hope, individuals may become demotivated and vulnerable to mental health problems [59].

This study utilized a cross-sectional design, which limited its ability to establish causal relationships. The data were collected using self-administered questionnaires, which may be subject to recall bias or social desirability bias, particularly in sensitive areas such as sexual behaviors, negative experiences, and mental health symptoms. The non-probability sampling and online data collection methods used may further limit the generalizability of the findings to the broader MSM population in urban Thailand. Due to the nature of online recruitment, the sample may have been skewed toward younger, more educated individuals with better digital access and higher socioeconomic status. However, this approach may be appropriate given the study’s focus on a specific population that faces barriers to disclosure and participation, particularly when addressing sensitive issues. The assessments were conducted using validated screening tools, which indicate risk levels rather than provide clinical diagnoses. Consequently, the reported prevalence of psychological distress and depression may differ from clinically diagnosed rates. Although adjustments were made for demographic and health-related covariates in the regression analyses, there remain potential unmeasured confounding variables, such as the intensity of minority stress, trauma history, and interpersonal factors, which could influence both DA use and mental health outcomes.

Thus, future research should employ a longitudinal study design to clarify causality and use qualitative approaches to explore user experiences and contexts influencing mental well-being. Investigating specific dating app features such as design, user culture, and matching algorithms could provide further insight into their impact on mental health. Expanding to other mental health outcomes like burnout, stress, anxiety, and self-esteem could also offer a more comprehensive overview of the effects of dating application use.

## 5. Conclusions

This study provides important insights into the DA use among MSM residing in urban areas of Thailand, revealing a high prevalence of DA usage, psychological distress, and probable depression. While the overall history of DA use was not significantly associated with mental health outcomes, further analysis within the current user group revealed that certain usage patterns and negative experiences, such as frequent rejection, privacy violations, and late-night usage, were significantly linked to poorer mental health. These findings suggest that mental health risks are shaped less by whether individuals use DAs and more by how and why they use them.

The results highlight the need for public health interventions and inclusive digital platform design that consider the psychological vulnerability of marginalized populations like MSM. Integrating mental health screening and support into sexual health services can enable early detection and timely care. Education on safe app use, managing expectations, and building self-esteem is essential to reduce negative impacts. App developers should improve protective measures against harassment, abuse, and scams and also offer accessible mental health resources within their platforms. Additionally, promoting family and community acceptance of sexual identity is crucial for reducing stigma and supporting mental well-being. Together, these strategies could help improve the overall health outcomes for MSM and other sexual minorities.

## Figures and Tables

**Table 1:** Demographic and health-related information of participants, classified by mental health outcomes (*n* = 442).

Demographic and Health-Related Information	n (%)	Psychological Distress		*p*-Value	Probable Depression		*p*-Value
		No	Yes		No	Yes	
Total	442 (100.0)	300 (67.9)	142 (32.1)	-	294 (66.5)	148 (33.5)	-
Age				0.061			0.043 *
18–25 years	121 (27.4)	75 (62.0)	37 (38.0)		74 (61.2)	47 (38.8)	
26–35 years	240 (54.3)	162 (67.5)	78 (32.5)		157 (65.4)	83 (34.6)	
More than 35 years	81 (18.3)	63 (77.8)	18 (22.2)		63 (77.8)	18 (22.2)	
Diverse Sexuality and Gender				0.324			0.989
Cisgender male	30 (6.8)	21 (70.0)	9 (30.0)		20 (66.7)	10 (33.3)	
Gay	352 (79.6)	234 (66.5)	118 (33.5)		233 (66.2)	119 (33.8)	
Bisexual	26 (5.9)	19 (73.1)	7 (26.9)		17 (65.4)	9 (34.6)	
Transgender woman	31 (7.0)	25 (80.6)	6 (19.4)		22 (71.0)	9 (29.0)	
Queer	3 (0.7)	1 (33.3)	2 (66.7)		2 (66.7)	1 (33.3)	
Relationship status				0.252			0.742
Single	269 (60.9)	181 (67.3)	88 (32.7)		177 (65.8)	92 (34.2)	
Monogamous	135 (30.5)	97 (71.9)	38 (28.1)		93 (68.9)	42 (31.1)	
Polyamorous	38 (8.6)	22 (57.9)	16 (42.1)		24 (63.2)	14 (36.8)	
Education level				0.147			0.041 *
Below bachelor’s degree	101 (22.9)	66 (65.3)	35 (34.7)		57 (56.4)	44 (43.6)	
Bachelor’s degree	292 (66.1)	206 (70.5)	86 (29.5)		201 (68.8)	91 (31.2)	
Above bachelor’s degree	49 (11.0)	28 (57.1)	21 (42.9)		36 (73.5)	13 (26.5)	
Monthly income				0.366			0.319
≤THB 15,000	149 (33.7)	95 (63.8)	54 (36.2)		94 (63.1)	55 (36.9)	
THB 15,001–29,999	160 (36.2)	114 (71.3)	46 (28.7)		105 (65.6)	55 (34.4)	
≥THB 30,000	133 (30.1)	91 (68.4)	42 (31.6)		95 (71.4)	38 (28.6)	
Occupation				0.616			0.125
Unemployed	59 (13.3)	37 (62.7)	22 (37.3)		39 (66.1)	20 (33.9)	
Government employee	82 (18.6)	58 (70.7)	24 (29.3)		62 (75.6)	20 (24.4)	
Freelancer	40 (9.0)	29 (72.5)	11 (27.5)		21 (52.5)	19 (47.5)	
Self-employed	57 (12.9)	35 (61.4)	22 (38.6)		40 (70.2)	17 (29.8)	
Private sector employee	204 (46.2)	141 (69.1)	63 (30.9)		132 (64.7)	72 (35.3)	
Residential area				0.483			0.249
Bangkok	201 (45.5)	133 (66.2)	68 (33.8)		128 (63.7)	73 (36.3)	
Urban municipalities	241 (54.5)	167 (69.3)	74 (30.7)		166 (68.9)	75 (31.1)	
Family relationship				<0.001 *			0.030 *
Fair	84 (19.0)	42 (50.0)	42 (50.0)		46 (54.8)	38 (45.2)	
Good	155 (35.1)	110 (71.0)	45 (29.0)		104 (67.1)	51 (32.9)	
Very good	203 (45.9)	148 (72.9)	55 (27.1)		144 (70.9)	59 (29.1)	
Body image satisfaction				<0.001 *			<0.001 *
Fair	118 (26.7)	64 (54.2)	54 (45.8)		55 (46.6)	63 (53.4)	
Good	202 (45.7)	140 (69.3)	62 (30.7)		149 (73.8)	53 (26.2)	
Very good	122 (27.6)	96 (78.7)	26 (21.3)		90 (73.8)	32 (26.2)	
Physical health satisfaction				<0.001 *			0.001 *
Fair	130 (29.4)	73 (56.2)	57 (43.8)		71 (54.6)	59 (45.4)	
Good	199 (45.0)	135 (67.8)	64 (32.2)		135 (67.8)	64 (32.2)	
Very good	133 (25.6)	92 (81.4)	21 (18.6)		88 (77.9)	25 (22.1)	
Perception of social stigma				0.089			0.021 *
None	133 (30.1)	100 (75.2)	33 (24.8)		100 (75.)	33 (24.8)	
Low level	106 (24.0)	67 (63.2)	39 (36.8)		71 (67.0)	35 (33.0)	
High level	203 (45.9)	133 (65.5)	70 (34.5)		123 (60.6)	80 (39.4)	
Sexual activity history within the past 6 months				0.529			0.166
No	66 (14.9)	47 (71.2)	19 (28.8)		39 (59.1)	27 (40.9)	
Yes	376 (85.1)	253 (67.3)	123 (32.7)		255 (67.8)	121 (32.2)	
Number of sexual partners (*n* = 376)				0.045 *			0.810
Single sexual partner	140 (37.2)	103 (73.6)	37 (26.4)		96 (68.6)	44 (31.4)	
Multiple sexual partners	236 (62.8)	150 (63.6)	86 (36.4)		159 (67.4)	77 (32.6)	
Condom use (*n* = 376)				0.212			0.454
Always	135 (35.9)	100 (74.1)	35 (25.9)		98 (72.6)	37 (27.4)	
Almost every time	113 (30.1)	73 (64.6)	40 (35.4)		77 (68.1)	36 (31.9)	
Sometimes	71 (18.9)	46 (64.8)	25 (35.2)		45 (63.4)	26 (36.6)	
Rarely	24 (6.4)	16 (66.7)	8 (33.3)		16 (66.7)	8 (33.3)	
Never	33 (8.8)	18 (54.5)	15 (45.5)		19 (57.6)	14 (42.4)	
Group sex participation (*n* = 376)				0.005 *			0.139
No	201 (53.5)	148 (73.6)	53 (26.4)		143 (71.1)	58 (28.9)	
Yes	175 (46.5)	105 (60.0)	70 (40.0)		112 (64.0)	63 (36.0)	
Alcohol drinking before or during sex (*n* = 376)				0.448			0.063
No	197 (52.4)	136 (69.0)	61 (31.0)		142 (72.1)	55 (27.9)	
Yes	179 (47.6)	117 (65.4)	62 (34.6)		113 (63.1)	66 (36.9)	
Smoking before or during sex (*n* = 376)				0.299			0.002 *
No	299 (79.5)	205 (68.6)	94 (31.4)		214 (71.6)	85 (28.4)	
Yes	77 (20.5)	48 (62.3)	29 (37.7)		41 (53.2)	36 (46.8)	
Substance use before or during sex (*n* = 376)				0.081			0.398
No	328 (87.2)	226 (68.9)	102 (31.1)		225 (68.6)	103 (31.4)	
Yes	48 (12.8)	27 (56.3)	21 (43.8)		30 (62.5)	18 (37.5)	
Poppers use before or during sex (*n* = 376)				0.330			0.936
No	200 (53.2)	139 (69.5)	61 (30.5)		136 (68.0)	64 (32.0)	
Yes	176 (46.8)	114 (64.8)	62 (35.2)		119 (67.6)	57 (32.4)	
HIV testing within the past 6 months ^†^				0.224			0.088
No	140 (32.3)	101 (72.1)	39 (27.9)		102 (72.9)	38 (27.1)	
Yes	294 (67.7)	195 (66.3)	99 (33.7)		190 (64.6)	104 (35.4)	

* *p*-value < 0.05 ^†^ Missing data (*n* = 8).

**Table 2:** History of DA usage and comparison of mental health outcomes of participants.

Mental Health Outcome	All n (%)	History of Dating App Usage			
		Never	Ever Used	Current Use	*p*-Value
Total	442 (100.0)	54 (12.2)	111 (25.1)	277 (62.7)	-
GHQ-12 score					
Mean (SD)	1.6 (2.5)	1.6 (2.7)	1.6 (2.6)	1.6 (2.5)	0.946 ^†^
Min–Max	0–12	0–12	0–11	0–11	
Psychological distress, n (%)	142 (32.1)	16 (29.6)	34 (30.6)	92 (33.2)	0.811 ^‡^
PHQ-9 score					
Mean (SD)	6.5 (5.8)	5.8 (5.6)	6.4 (5.5)	6.7 (6.0)	0.660 ^†^
Min–Max	0–27	0–23	0–25	0–27	
Probable depression, n (%)	148 (33.5)	19 (35.2)	35 (31.5)	94 (33.9)	0.867 ^‡^

^†^ Kruskal–Wallis test, ^‡^ chi-square test.

**Table 3:** Patterns, motivations, and experiences related to dating application use and their associations with mental health outcomes (*n* = 277).

Dating Application Use	All *n* (%)	Psychological Distress					Probable Depression				
		*n* (%)	COR (95% CI)	*p*-Value	AOR (95% CI) ^†^	*p*-Value	*n* (%)	COR (95% CI)	*p*-Value	AOR (95% CI) ^‡^	*p*-Value
DA usage pattern											
Number of DAs used											
≤3 apps	126 (45.5)	33 (26.2)	1		1		40 (31.7)	1		1	
>3 apps	151 (54.5)	59 (39.1)	1.81 (1.08–3.02)	0.024 *	1.50 (0.87–2.59)	0.149	54 (35.8)	1.20 (0.73–1.98)	0.482	1.02 (0.59–1.79)	0.932
Duration since first use											
<2 years	61 (22.0)	18 (29.5)	1		1		20 (32.8)	1		1	
≥2 years	216 (78.0)	74 (34.3)	1.24 (0.67–2.31)	0.487	1.10 (0.57–2.13)	0.784	74 (34.3)	1.07 (0.58–1.95)	0.830	1.08 (0.54–2.14)	0.829
App usage frequency											
<Monthly	30 (10.8)	11 (36.7)	1		1		9 (30.0)	1		1	
Monthly	41 (14.8)	13 (31.7)	0.80 (0.30–2.16)	0.663	0.68 (0.23–2.03)	0.494	16 (39.0)	1.49 (0.55–4.07)	0.433	1.21 (0.40–3.63)	0.736
Weekly	86 (31.0)	25 (29.1)	0.71 (0.29–1.70)	0.440	0.59 (0.23–1.53)	0.279	31 (36.0)	1.32 (0.54–3.22)	0.549	1.08 (0.41–2.89)	0.872
Daily	120 (43.3)	43 (35.8)	0.96 (0.42–2.21)	0.932	0.88 (0.35–2.19)	0.779	38 (31.7)	1.08 (0.45–2.58)	0.860	0.91 (0.35–2.32)	0.839
Duration per session											
<20 min	194 (70.0)	68 (35.1)	1		–		62 (32.0)	1		1	
≥20 min	83 (30.0)	24 (28.9)	0.75 (0.43–1.32)	0.321	0.70 (0.39–1.27)	0.243	32 (38.6)	1.34 (0.78–2.28)	0.289	1.50 (0.82–2.75)	0.192
App use days											
Every day	187 (67.5)	66 (35.3)	1		1		63 (33.7)	1		1	
Friday–Sunday only	90 (32.5)	26 (28.9)	0.74 (0.43–1.29)	0.290	0.83 (0.46–1.49)	0.537	31 (34.4)	1.03 (0.61–1.76)	0.901	1.29 (0.72–2.33)	0.389
App usage during late-night hours (02:00–06:00)											
No	217 (78.3)	64 (29.5)	1		1		69 (31.8)	1		1	
Yes	60 (21.7)	28 (46.7)	2.09 (1.17–3.76)	0.013 *	2.02 (1.08–3.78)	0.028 *	25 (41.7)	1.53 (0.85–2.76)	0.155	1.29 (0.67–2.46)	0.444
Motivation for DA use											
Making friends											
No	60 (21.7)	16 (26.7)	1		1		15 (25.0)	1		1	
Yes	217 (78.3)	76 (35.0)	1.48 (0.78–2.80)	0.226	1.67 (0.84–3.31)	0.143	79 (36.4)	1.72 (0.90–3.28)	0.101	1.85 (0.91–3.77)	0.088
Relieving loneliness											
No	49 (17.7)	14 (28.6)	1		1		11 (22.4)	1		1	
Yes	228 (82.3)	78 (34.2)	1.30 (0.66–2.56)	0.448	1.49 (0.73–3.06)	0.275	83 (36.4)	1.98 (0.96–4.08)	0.065	2.05 (0.93–4.53)	0.076
Hooking up											
No	57 (20.6)	12 (21.1)	1		1		12 (21.1)	1		1	
Yes	220 (79.4)	80 (36.4)	2.14 (1.07–4.29)	0.031 *	1.75 (0.81–3.76)	0.152	82 (37.3)	2.23 (1.11–4.46)	0.023 *	2.27 (1.05–4.93)	0.037 *
Finding a long-term relationship											
No	102 (36.8)	31 (30.4)	1		1		26 (25.5)	1		1	
Yes	175 (63.2)	61 (34.9)	1.23 (0.73–2.07)	0.447	1.18 (0.67–2.09)	0.559	68 (38.9)	1.86 (1.08–3.19)	0.024 *	1.82 (0.99–3.35)	0.053
Using sexual services											
No	228 (82.3)	73 (32.0)	1		1		71 (31.1)	1		1	
Yes	49 (17.7)	19 (38.3)	1.34 (0.71–2.55)	0.363	1.36 (0.67–2.78)	0.399	23 (46.9)	1.96 (1.04–3.66)	0.036 *	1.84 (0.87–3.86)	0.109
Using other services											
No	232 (83.8)	73 (31.5)	1		1		76 (32.8)	1		1	
Yes	45 (16.2)	19 (42.2)	1.59 (0.83–3.06)	0.163	1.54 (0.75–3.16)	0.236	18 (40.0)	1.37 (0.71–2.64)	0.349	1.41 (0.66–2.98)	0.372
Killing time											
No	96 (34.7)	27 (28.1)	1		1		36 (37.5)	1		1	
Yes	181 (65.3)	65 (35.9)	1.43 (0.84–2.45)	0.191	1.43 (0.81–2.55)	0.219	58 (32.0)	0.79 (0.47–1.32)	0.362	0.80 (0.45–1.42)	0.444
Experience related to DA use											
Offline meeting disappointment											
Infrequently ^§^	198 (71.5)	60 (30.3)	1		1		70 (35.3)	1		1	
Frequently	79 (28.5)	32 (40.5)	1.57 (0.91–2.69)	0.105	1.23 (0.68–2.23)	0.491	24 (30.4)	0.80 (0.46–1.40)	0.430	0.55 (0.28–1.08)	0.081
Matching failure											
Infrequently	149 (53.8)	37 (24.8)	1		1		40 (28.2)	1		1	
Frequently	128 (46.2)	55 (43.0)	2.28 (1.37–3.80)	0.002 *	1.95 (1.12–3.38)	0.017 *	52 (40.6)	1.74 (1.06–2.88)	0.030 *	1.37 (0.78–2.42)	0.272
Ignoring/Rejection											
Infrequently	163 (58.8)	42 (25.8)	1		1		48 (29.4)	1		1	
Frequently	114 (41.2)	50 (48.9)	2.25 (1.35–3.75)	0.002 *	2.07 (0.18–3.62)	0.011 *	46 (40.3)	1.62 (0.98–2.68)	0.060	1.36 (0.75–2.45)	0.309
Ghosting											
Infrequently	138 (49.8)	35 (25.4)	1		1		43 (31.2)	1		1	
Frequently	139 (50.2)	57 (41.0)	2.05 (1.23–3.41)	0.006 *	1.78 (1.02–3.11)	0.043 *	51 (36.7)	1.28 (0.78–2.11)	0.331	1.08 (0.61–1.93)	0.784
Privacy violation											
Never	87 (31.4)	25 (28.7)	1		1		17 (19.5)	1		1	
Ever	190 (68.6)	67 (35.3)	1.35 (0.78–2.35)	0.285	1.21 (0.67–2.18)	0.531	77 (40.5)	2.81 (1.54–2.13)	0.001 *	2.76 (1.42–5.38)	0.003 *
Unsolicited sexual images											
Never	107 (38.6)	30 (28.0)	1		1		27 (25.2)	1		1	
Ever	170 (61.4)	62 (36.5)	1.47 (0.87–2.50)	0.148	1.46 (0.83–2.54)	0.188	67 (39.4)	1.93 (1.13–3.29)	0.016 *	2.04 (1.11–3.74)	0.021 *
Physical assault											
Never	176 (63.5)	57 (32.4)	1		1		46 (26.1)	1		1	
Ever	101 (36.5)	35 (34.7)	1.11 (0.66–1.86)	0.700	1.11 (0.63–1.96)	0.714	48 (47.5)	2.56 (1.53–4.29)	<0.001 *	2.97 (1.57–5.61)	0.001 *
Sexual harassment											
Never	163 (58.8)	55 (33.7)	1		1		44 (27.0)	1		1	
Ever	114 (41.2)	37 (32.5)	0.94 (0.57–1.57)	0.823	0.90 (0.52–1.57)	0.713	50 (43.9)	2.11 (1.27–3.51)	0.004 *	2.54 (1.37–4.70)	0.003 *
Money scam											
Never	140 (50.5)	44 (31.4)	1		1		36 (25.7)	1		1	
Ever	137 (49.5)	48 (35.0)	1.18 (0.71–1.94)	0.524	1.15 (0.68–1.97)	0.602	58 (42.3)	2.12 (1.28–3.53)	0.004 *	2.59 (1.41–4.77)	0.002 *
Overall disappointment											
None	48 (17.3)	15 (31.3)	1		1		10 (20.8)	1		1	
Mild	50 (18.1)	13 (26.0)	0.77 (0.32–1.86)	0.566	0.75 (0.29–1.92)	0.545	14 (28.0)	1.48 (0.58–3.75)	0.411	1.76 (0.62–4.96)	0.285
Moderate	87 (31.4)	26 (29.9)	0.94 (0.44–2.01)	0.869	0.84 (0.37–1.89)	0.670	30 (34.5)	2.00 (0.88–4.56)	0.100	2.08 (0.83–5.19)	0.118
High	67 (24.2)	25 (37.3)	1.31 (0.60–2.87)	0.501	1.27 (0.54–2.97)	0.581	25 (37.3)	2.26 (0.96–5.32)	0.061	2.71 (1.03–7.13)	0.043 *
Extreme	25 (9.0)	13 (52.0)	2.38 (0.88–6.44)	0.087	2.09 (0.72–6.03)	0.174	15 (60.0)	5.70 (1.97–16.47)	0.001 *	5.98 (1.84–19.41)	0.003 *

* *p*-value < 0.05. ^†^ Adjusted for family relationship, body satisfaction, physical health satisfaction, number of sexual partners, and group sex participation. ^‡^ Adjusted for age, education, family relationship, body satisfaction, physical health satisfaction, level of perceived social stigma, and smoking before or during sex. ^§^ ‘Infrequently’ included responses of ‘never’, ‘rarely’, and ‘sometimes’, while ‘frequently’ included ‘often’ and ‘always’.

## Data Availability

The data are not publicly available due to privacy and ethical reasons.
